# Smartphone Addiction in Substance Use Patients: Who Is Most Vulnerable?

**DOI:** 10.1192/j.eurpsy.2026.10907

**Published:** 2026-07-31

**Authors:** E. M. Bahri, J. Nasri, S. Hleli, M. Bennsir, W. Abdelghaffar

**Affiliations:** Hope Department for Addiction Care, Jbel Ouest Health Center, Zaghouan, Tunisia

## Abstract

**Introduction:**

Smartphone addiction is an emerging behavioral disorder. Its comorbidity with Substance Use Disorders is poorly understood, potentially creating a complex clinical profile.

**Objectives:**

This study aims to describe the sociodemographic and clinical characteristics of substance use disorder patients with co-occurring smartphone addiction to identify who is most vulnerable.

**Methods:**

A cross-sectional study was conducted on 60 patients diagnosed with substance use disorder recruited from the Hope Department for Addiction Care at the Jbel Ouest Health Center. Participants completed a sociodemographic questionnaire and the Smartphone Addiction Scale-Short Version (SAS-SV) in Arabic. Based on established thresholds (≥31 for males, ≥33 for females), patients were categorized into two groups: with smartphone addcition (SAS-SV+) and without smartphone addicition (SAS-SV-). Data from clinical records were also collected. Data was collected and analyzed using IBM SPSS Statistics software, version 27.

**Results:**

Results for the SAS-SV+ Group (n=23): Patients with smartphone addiction had a mean age of 30.0 years (SD=9.0, range: 19-52) and were exclusively male (n=23, 100%). The majority were single (n=15, 65.2%) with a secondary education level (n=17, 73.9%). The most common employment status was day labourer (n=9, 39.1%). A significant minority had a personal psychiatric diagnosis (n=6, 26.1%), with depression being the most reported (n=4, 17.4%). Notably, this group showed high rates of substance use, with cannabis (n=13, 56.5%) and cocaine/crack (n=10, 43.5%) being the most prevalent. Furthermore, a family history of addiction was reported by 39.1% (n=9) of this group.

**Conclusions:**

A significant proportion of substance use disorder patients present with comorbid smartphone addiction, characterized by a distinct sociodemographic and clinical profile. Recognizing this profile is crucial for clinicians to screen for digital addiction and develop integrated treatment strategies that address both chemical and behavioral dependencies.

**Disclosure of Interest:**

None Declared

